# Jenner, Pasteur, and Koch

**Published:** 1890-12-13

**Authors:** 


### The Doctor's Armchair.

### JENNER, PASTEUR, AND KOCH.

Jenner, Pasteur, and Koch, all belong to the class of inoculators; but they are inoculators with divergences. Inoculation, at the present time, is rich in promise; and it is well that the attention of medical men and others should be concentrated upon it. The clear appreciation of the differences between Jenner's and Pasteur's inoculations, and between Pasteur's and Koch's, is of considerable help in giving shape and definiteness to our mental concepts on the subject. And such definiteness of conception is all important with regard both to the present use of inoculation, and to its future extension. We are all familiar with the practice of vaccination, but familiar in a routine way. It must, however, be borne in mind that vaccination cannot yet be considered to have the authority of venerable age. "Art is long, but life is short," and many are apt to think that what was established before their own birth, must of necessity be venerable. But those who are disposed to attribute finality to vaccination, should remember how many centuries the Ptolemaic system of astronomy held sway before it was replaced by the Copernican; and how many more centuries still rolled by before Newton gave correctness and authority to the guessings and half demonstrations of the Polish philosopher. At this very moment, when the world is being filled with the name and the doings of Koch the latest of the inoculators, Crookshank and Creighton, both excellent workers in the Bacteriological field, are not only questioning, but denying that there is any value whatever in vaccination, or any soundness at all in the theories of Jenner, the inoculator par excellence. This is well worth thinking about; but our present purpose does not permit us to do more than mention it.

A comparison of the objects which the three inoculators, whose names we have placed at the head of this article, have in view is our present purpose. A comparison of the objects, not of the methods, for the methods are practically identical. Why did Jenner begin to vaccinate? Because he had heard, over and over again in his country experience that during epidemics of small-pox certain persons escaped the disease. Who were those persons, and why did they escape? They were men and women whose work was among cattle, and the reason why they escaped was popularly believed to be because they had, at some time or other in their lives, accidentally taken the disease of cow-pox directly from the cow. Acting upon this hint Jenner reasoned that the virus of cow-pox was a preventive of small-pox; and so he took virus from cow-pox and inoculated the human subject with it experimentally. The result we know, or we think we know, Crookshank and Creighton notwithstanding. For if statistics alone can form a safe basis of demonstration, vaccination is proved to be one of the greatest of all discoveries made by scientific man.

What we wish, however, more particularly now to concentrate attention upon is the peculiar object the vaccinator has in view when he inoculates a person with bovine or human lymph. His object is not to cure a disease already established and active in the system, but to prevent the possibility of the establishment of a particular disease, viz., small-pox. How this is done we need not now explain in detail. This much, however, may be said, that a great change is probably made in the blood, without in any way diminishing its nutritive power. A certain element in it which appears to be necessary for the development of small-pox seems to be impaired or destroyed by vaccination. That this theory is the true one is rendered very probable by the apparently indisputable fact that the immunity from small-pox increases directly with the number and pronounced character of the vaccine vesicles. The idea then in vaccination is to destroy the pabulum in the human system which is essential to the development of small-pox. This is prophylaxis, pure and simple.

The next important inoculator in the order of time is Pasteur. What concerns us most at the present moment is the object the Pasteurian has in view when he inoculates for hydrophobia. That object is quite different from Jenner's, inasmuch as it is a treatment for a disease already in the system, either latent or active. It is true that the Pasteurians look upon their method as prophylactic rather than curative. But the difficulties associated with such a hypothesis are so great as to be practically insurmountable. If Pasteur's treatment be really and truly a cure for hydrophobia, it can only be so in one of three ways—it must be either, like vaccination, destroy the pabulum which is necessary for the development of the hydrophobia virus; or it must destroy or render harmless the hydrophobia virus itself; or it must cut short and cure hydrophobic disease when established and in full activity. Which of these three results is brought about by Pasteur's inoculation, or whether any of them, it is not our purpose here to discuss. What we wish to point out is that Pasteur's object in inoculation is not, like Jenner's, purely prophylactic, but curative, though the *modus operandi* of the cure is still in the hypothetical stage, and the reality of the cure itself, in many important quarters, is vigorously denied.

The object of Koch in his inoculations is different from that of either Pasteur, or Jenner. It is not only curative; but curative in a particular way. Koch injects a fluid into the system, which is said to be endowed with the peculiar power of seeking out a particular diseased tissue in the body, and of destroying it. Like a bloodhound put upon the trail of a thief or a murderer, this fluid pays no heed to anything which may cross the trail of its enemy, or strive to divert it to side issues; it goes straight to its destination and destroys its victim, without hesitation or remorse. This is a fact practically without parallel in the long and varied history of man. Our object, however, at the present is not so much to discuss a thing so strange and new, but to point out that though inoculation be the method of Jenner, Pasteur, and Koch alike, yet the aim of the inoculation is in each case widely different; so different indeed that the three treatments though apparently identical, are yet in their essence as divergent as is the subcutaneous injection of morphia from the insertion of vaccine lymph, into the skin of the arm.

Two conclusions we wish to draw from this reasoning; first, that the Pasteurians are not entitled to claim any support for their theory from Jenner and the vaccinationists. Pasteur's treatment for hydrophobia stands or falls by the evidence which it can bring to its own support. The second conclusion is that if Pasteur can claim no support from Jenner, still less can Koch. There is not a suggestion of prophylaxis in Koch's inoculation for tuberculosis and lupus from beginning to end. In saying this, we are not so much as hinting a doubt of the efficacy of the new treatment. But we are urging and insisting that it must stand on its own merits, and on its own merits alone. In its essence it is a "specific" treatment, and being that and nothing else, it must be judged by the severest standards which medical science can apply. If, after the application of those standards, this specific treatment holds its ground, then, indeed, will Koch be proved worthy of the deepest admiration of all men in all time.

